# Droplet digital RT-PCR method for SARS-CoV-2 variants detection in clinical and wastewater samples

**DOI:** 10.3389/fmicb.2025.1635733

**Published:** 2025-07-03

**Authors:** Feng Wang, Yi Sun, Liming Gong, Lingxuan Su, Biaofeng Zhou, Xiuyu Lou, Yin Chen, Wen Shi, Haiyan Mao, Yanjun Zhang

**Affiliations:** Zhejiang Key Laboratory of Public Health Detection and Pathogenesis Research, Department of Microbiology, Zhejiang Provincial Center for Disease Control and Prevention (Zhejiang CDC), Hangzhou, China

**Keywords:** droplet digital RT-PCR, SARS-CoV-2 variants, clinical, wastewater, sensitivity, specificity, repeatability

## Abstract

**Objective:**

To establish a sensitive, specific, and precise quantitative detection method for SARS-CoV-2 variants using droplet digital RT-PCR (RT-ddPCR).

**Methods:**

Dual primer-probe sets targeting the SARS-CoV-2 nucleocapsid (N) and spike (S) genes were designed. The annealing temperature for RT-ddPCR was optimized using a gradient PCR system. The sensitivity, defined as the limit of detection (LOD), was determined by serially diluting SARS-CoV-2 RNA. The specificity of the RT-ddPCR assay was evaluated using SARS-CoV-2 variants and common respiratory viruses. Precision and repeatability were assessed by quantitatively repeating the detection on serial dilutions of SARS-CoV-2 RNA. Additionally, the results of RT-ddPCR for clinical and environmental wastewater samples were compared with those from RT-qPCR.

**Results:**

The optimal annealing temperature was 53.5°C. The LOD for the N and S genes of the original SARS-CoV-2 strain was 4.26 (95% CI: 3.12–9.89) and 3.87 (95% CI: 2.77–7.75) copies/reaction. The Delta strain exhibited LODs of 4.65 (N gene, 95% CI: 3.28–9.64) and 6.12 (S gene, 95% CI: 4.33–15.59) copies/reaction. The Omicron showed 4.07 (N gene, 95% CI: 3.11–6.26) and 4.58 (S gene, 95% CI: 3.43–7.40) copies/reaction. Importantly, the RT-ddPCR assay was repeatable with a coefficient of variation of less than 10% when RNA concentrations of SARS-CoV-2 were between 73.50 and 7,500 copies/reaction. The high specificity of the RT-ddPCR assay was demonstrated by its ability to correctly detect the thirty SARS-CoV-2 variants, while not other common respiratory viruses. For 148 clinical pharyngeal swab specimens, the positive rate for both RT-ddPCR and RT-qPCR was 86.49%, and a coincidence rate of 98.65% and a Kappa value of 0.94. Quantitative comparison of RT-ddPCR and RT-qPCR in 50 wastewater samples with low viral load, RT-ddPCR assay detected 50 positives for dual gene targets (N and S genes), whereas RT-qPCR assay only 21 exhibited concurrent positivity for dual gene targets, while 25 showed S gene detection, and 4 were negative for dual gene targets, suggesting our RT-ddPCR assay enabled absolute quantification of SARS-CoV-2 variants with low viral load.

**Conclusion:**

The RT-ddPCR assay developed in this study can be used for SARS-CoV-2 variants detection and quantitative analysis of clinical and environmental samples.

## 1 Introduction

As of May 2025, more than 777 million confirmed cases of coronavirus disease 2019 (COVID-19), caused by infection with severe acute respiratory syndrome coronavirus 2 (SARS-CoV-2), were reported (WHO, 2025), thus substantially affecting human health and global economic growth. SARS-CoV-2, a member of the *β*-coronavirus family, has an envelope and a non-segmented, positive-sense single-stranded RNA genome. It shares 79 and 50% genome sequence similarity with SARS-CoV and MERS-CoV, respectively (Lu et al., 2020; Rabaan et al., 2020). According to the World Health Organization (WHO), real-time fluorescence quantitative polymerase chain reaction (RT-qPCR) is the most frequently used method for diagnosing SARS-CoV-2 infection. However, the RT-qPCR assay has limited sensitivity, is vulnerable to variables such as standard curves, and is prone to false negatives in samples with low viral loads, thus hindering the prevention and management of outbreaks (Safiabadi Tali et al., 2021).

Droplet digital RT-PCR (RT-ddPCR) is based on the principle of limiting dilution PCR, with the PCR reaction mixture is uniformly partitioned into tens of thousands of independent micro-droplets. Consequently, some micro-droplets contain one or more template copies, whereas others lack the template. Each micro-droplet is subjected to PCR independently. A significant increase in fluorescence signal is detected for droplets containing template nucleic acid, whereas droplets lacking template nucleic acid maintain the background fluorescence intensity. Micro-droplets were dichotomously classified as positive or negative based on fluorescence signal thresholds (Xu et al., 2023). Finally, on the basis of the Poisson distribution, the number of positive micro-droplets is converted into a nucleic acid copy number, thus enabling absolute quantification of the target nucleic acid (Kojabad et al., 2021). Compared with RT-qPCR, RT-ddPCR exhibits higher sensitivity for samples with low viral load and stronger detection specificity. The RT-ddPCR method has been applied in the quantitative analysis of viruses such as hepatitis B virus, human immunodeficiency virus, Zika virus, enterovirus, parechovirus, and herpes simplex virus types 1 and 2 (Urso et al., 2016; Hui et al., 2018; Hayashi et al., 2022; Zhu et al., 2022).

The objective of this study was to establish an RT-ddPCR method for SARS-CoV-2 variants detection with high efficiency, specificity, and sensitivity. This method aims to improve detection accuracy in clinical and wastewater settings, decrease the occurrence of false-negative results, mitigate potential transmission risks, and contribute to more effective diagnostic strategies for SARS-CoV-2 variants infections (Ishak et al., 2021).

## 2 Materials and methods

### 2.1 Strains and samples

The original strain of SARS-CoV-2 and variants used for sensitivity evaluation were obtained from the laboratory of the Zhejiang Provincial Center for Disease Control and Prevention (Zhejiang CDC), China. The original strain, SARS-CoV-2/E6/WGF/2020/ZJ8, had a titer of 3.76 × 10^6^ TCID50/mL; the Delta strain, SARS-CoV-2/Vero/LXG/2021/ZJ28, had a titer of 3.73 × 10^5^ TCID50/mL; and the Omicron strain, SARS-CoV-2/E6/Gabriol/2022/ZJ60, had a titer of 3.16 × 10^5^ TCID50/mL.

Seven common respiratory viruses and SARS-CoV-2 pseudovirus quantification reference material (Fantasiabio, Zhejiang, China; RFKSS001) were used to evaluate the specificity of RT-ddPCR. Seven common respiratory viruses were obtained from Zhejiang CDC, including influenza A (H1N1)pdm 09, Victoria lineage of influenza B virus, respiratory syncytial virus subtype A, human parainfluenza virus type III, adenovirus type 7, human coronavirus OC43, and human coronavirus 229E. The original strain and thirty SARS-CoV-2 variants, including the three variants of concern (VOCs) (Alpha, Delta, and Omicron) were used for specificity evaluation. The Omicron variants comprised 28 sublineages: BA.1.1, BA.2.12.1, BA.2.3, XBB.1.5.4, XBB.1.9.2, EG.5.1, EG.5.1.1, HK.3, XBB.1.16, FU.1, XBB.1.22, BA.4.1, BA.5.2, BF.7, BA.5.2.48, DY.2, BQ.1.1, BF.7.14, JN.1, JN.1.4.5, LB.1.2, JN.1.16, KP.2, KP.3.1.1, JN.1.67.1, XDV.1, XDV.1.5, and NB.1.

For quantitative comparison between RT-ddPCR and RT-qPCR, a total of 148 nasopharyngeal samples collected from fever clinics between June 2022 and December 2024 at three hospitals: Yiwu Central Hospital, Hangzhou First People’s Hospital, and Children’s Hospital, Zhejiang University School of Medicine, China. All samples were collected in 3 mL universal viral transport medium and transported to the laboratory immediately or storage at −80°C until use. Additionally, 50 environmental wastewater samples were obtained from the inlet of the Qianjiang sewage-treatment plant in Xiaoshan District, Hangzhou City, Zhejiang Province, China. The initial volume of each wastewater sample was 5 L.

### 2.2 Wastewater preparation

The samples were transported to the laboratory at 4°C and concentrated through hyperfiltration. Firstly, 400 mL of the 5 L untreated wastewater was centrifuged at 2,500 × *g* for 20 min at 4°C after blending. The liquid supernatant was then placed in an aseptic bottle and concentrated with a tubular ultrafiltration membrane connected to an ultrafiltration device. Next, the viruses retained on the ultrafiltration membrane were eluted with 2 mL of 3% beef extract solution. The eluates were stored at −80°C until use.

### 2.3 RNA extraction

RNeasy Mini Kit (Qiagen, Hilden, Germany; 74,104) was used to extract RNA according to the manufacturer’s instructions. A total of 50 μL viral RNA was extracted from 200 μL clinical and concentrated wastewater samples. The RNA was stored at −80°C until use.

### 2.4 Primer and probe sets

SARS-CoV-2 specific primer-probe sets targeting the N and S genes were developed for RT-ddPCR and RT-qPCR are provided in Table 1. The primer-probe sets were synthesized by Shanghai Sangon Company (Shanghai, China). All primers and probes were prepared at a concentration of 20 μmol/L, and primer-probe mix of the N and S genes were prepared in working solutions with a volume of 2:2:1.

**Table 1:** SARS-CoV-2 primer-probes used in this study.

Target gene	Type	Sequence (5′-3′)
N gene	Forward primer	ACATTGGCACCCGCAATCC
	Reverse primer	GCTTGACTGCCGCCTCTGCT
	Probe	FAM-5’-CGTGCTACAACTTCCTCAAGGAACA-3’-BHQ1
S gene	Forward primer	TTGATCACAGGCAGACTTCAAAGT
	Reverse primer	AGCTCTGATTTCTGCAGCTCTAATT
	Probe	VIC-5’-TGCAGACATATGTGACTCA-3’-BHQ1

### 2.5 RT-ddPCR for SARS-CoV-2 quantification

Absolute quantification of SARS-CoV-2 RNA was performed with the One-Step RT-ddPCR Advanced Kit for Probes (Bio-Rad, Hercules, USA; 1,864,021). The reaction mixture volume of 20 μL comprised 5 μL Supermix, 2 μL primer-probe mix, 2 μL reverse transcriptase, 1 μL 300 mM dithiothreitol (DTT), 8 μL nuclease-free water, and 2 μL RNA template. According to the manufacturer’s guidelines, the reaction mixture was used to produce droplets with a Bio-Rad Auto Droplet Generator (Bio-Rad, USA). Thermal cycling for all RT-ddPCR assays was performed with a T100 Thermal Cycler (Bio-Rad, USA) under the following amplification conditions: 45°C for 60 min; 95°C for 10 min; 40 cycles of 95°C for 30 s and 52°C to 60°C for 1 min; 98°C for 10 min; and storage at 4°C. Droplet signals were read in different channels using the QX200 Droplet Reader (Bio-Rad, USA), FAM channel for the N gene, and VIC channel for the S gene. Data were considered valid if the total number of droplets in each tube was ≥ 10,000. A sample was considered positive if the number of droplets exceeded three and negative if the number of droplets was three or fewer. Results are expressed as copies per reaction (copies/reaction).

### 2.6 Optimization of annealing temperature and DTT concentration for RT-ddPCR

We used the original strain to optimize the RT-ddPCR annealing temperature and DTT concentration. The annealing temperature of RT-ddPCR was optimized by testing eight temperatures (60.0, 59.4, 58.4, 56.9, 55.1, 53.5, 52.5, and 52.0°C) with the described method. The optimal annealing temperature was determined on the basis of the signal discrimination and nucleic acid copy number of each reaction.

In PCR systems, DTT is frequently employed as a protein reducing agent to preserve the sulfhydryl group of cysteine in proteins in a reduced state and to safeguard PCR reaction enzymes. Subsequently, with the optimal annealing temperature, a 300 mM DTT concentration in the RT-ddPCR reaction system was optimized. Two concentrations, 0.5 μL and 1 μL of 300 mM DTT, were tested in four repeated experiments, and the results were analyzed to determine the optimal reaction conditions.

### 2.7 RT-qPCR reaction system

An AgPath-ID™ One-step RT-PCR Kit (Thermo Fisher, Carlsbad, CA, USA; AM1005) was used to prepare a 25 μL RT-qPCR reaction system comprising 12.5 μL 2 × RT-PCR buffer, 1.5 μL primer-probe mix, 1 μL reverse transcriptase, 8 μL nuclease-free water, and 2 μL RNA template. The RT-qPCR reaction conditions were as follows: 45°C for 10 min; 95°C for 10 min; and 40 cycles of 94°C for 15 s and 53.5°C for 35 s. The cycle threshold (CT) value was derived from the amplification of the N gene and S gene of SARS-CoV-2 variants using the ABI 7500 Real Time PCR System (Applied Biosystems, USA).

### 2.8 Evaluation of RT-ddPCR sensitivity, specificity, and repeatability

We assessed the sensitivity of the RT-ddPCR method by determining the LOD for three strains of SARS-CoV-2 (original, Delta, and Omicron). The initial nucleic acid concentrations of these strains were quantified with identical primer-probe systems in a preliminary experiment. Samples were initially diluted by a factor of 10 and subsequently 2-fold serially diluted for samples with low nucleic acid concentrations. The diluted nucleic acid samples underwent RT-ddPCR detection to determine the positive detection rate. LOD was defined as the concentration corresponding to the 95% confidence interval of nucleic acid copies. A lower LOD indicated higher detection sensitivity.

Additionally, influenza A (H1N1)pdm 09, Victoria lineage of influenza B virus, respiratory syncytial virus subtype A, parainfluenza virus type III, adenovirus type 7, human coronavirus OC43, human coronavirus 229E, and SARS-CoV-2 pseudovirus quantification reference material were used to evaluate the specificity of the RT-ddPCR method developed in this study.

SARS-CoV-2 pseudovirus nucleic acid standards were utilized to extract nucleic acids and evaluate the repeatability of RT-ddPCR. The concentrations of positive quantitative reference materials ranged from 1 × 10^2^ to 1 × 10^6^ copies/mL, accompanied by a negative control group. Each concentration group underwent 16 tests under optimal RT-ddPCR conditions. The resulting data were used to calculate the mean, standard deviation, and coefficient of variation to assess the repeatability of the experiment. A coefficient of variation less than 10% indicated good repeatability.

### 2.9 Comparison of RT-ddPCR and RT-qPCR for quantitative detection of SARS-CoV-2 variants

The original strain and thirty variants of SARS-CoV-2 were selected: Alpha (B.1.1.7), Delta (B.1.617.2), BA.1.1, BA.2.12.1, BA.2.3, XBB.1.5.4, XBB.1.9.2, EG.5.1, EG.5.1.1, HK.3, XBB.1.16, FU.1, XBB.1.22, BA.4.1, BA.5.2, BF.7, BA.5.2.48, DY.2, BQ.1.1, BF.7.14, JN.1, JN.1.4.5, LB.1.2, JN.1.16, KP.2, KP.3.1.1, JN.1.67.1, XDV.1, XDV.1.5, and NB.1. The RT-ddPCR and RT-qPCR methods were used for quantitative detection, and reaction systems were as previously described.

### 2.10 Comparison of RT-ddPCR and RT-qPCR for quantitative detection in clinical and wastewater samples

A total of 148 clinical specimens from three hospitals (Yiwu Central Hospital, Hangzhou First People’s Hospital, and Children’s Hospital, Zhejiang University School of Medicine, China) were analyzed with the RT-ddPCR method described previously. Additionally, 50 wastewater samples from a sewage treatment plant in Hangzhou underwent the same extraction and amplification methods. The results obtained via RT-ddPCR subsequently compared with those obtained via RT-qPCR.

### 2.11 Statistical analysis

RT-ddPCR data were analyzed using QuantSoft 1.7.4.0917 software (Bio-Rad). The LOD, the coincidence rate, and Kappa value were calculated through SPSS 26.0 software, with a threshold for positive detection set at 95%. Statistical significance was set at *p* < 0.05.

## 3 Results

### 3.1 Determination of the optimal annealing temperature and DTT concentration for RT-ddPCR

**Figure 1:**
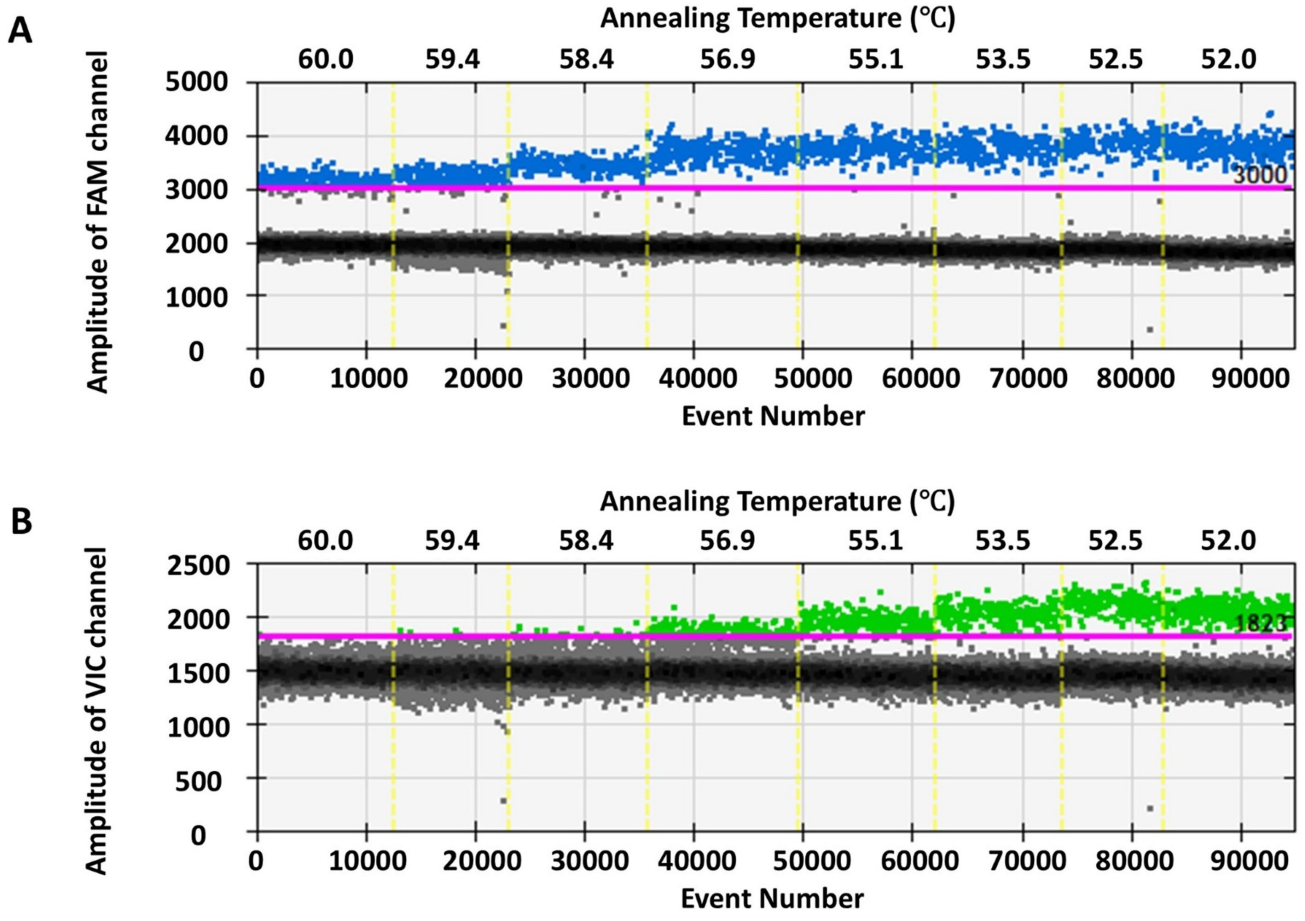
One-dimensional scatter plot of RT-ddPCR assay for SARS-CoV-2 N **(A)** and S **(B)** genes at various annealing temperatures. The x-axis in panels **(A,B)** represents different amplification annealing temperatures, while the y-axis in panels **(A,B)** represents the fluorescence amplitude in FAM and VIC channels, respectively.

Eight gradient annealing temperatures (60.0, 59.4, 58.4, 56.9, 55.1, 53.5, 52.5, and 52.0°C) were tested, revealing that as the temperature decreases, the RT-ddPCR amplification efficiency gradually increases (see Table 2). Improved separation between positive and negative droplets was notably observed within the range of 53.5–52.0°C (see Figure 1). To ensure PCR specificity and optimize amplification efficiency, we identified 53.5°C as the optimal annealing temperature.

**Table 2:** Copy numbers of SARS-CoV-2 N and S genes at various annealing temperatures, detected by RT-ddPCR.

Temperature (°C)	N gene (copies/reaction)	S gene (copies/reaction)
60.0	552	11
59.4	504	24
58.4	502	68
56.9	534	410
55.1	520	576
53.5	530	534
52.5	504	570
52.0	670	746

**Figure 2:**
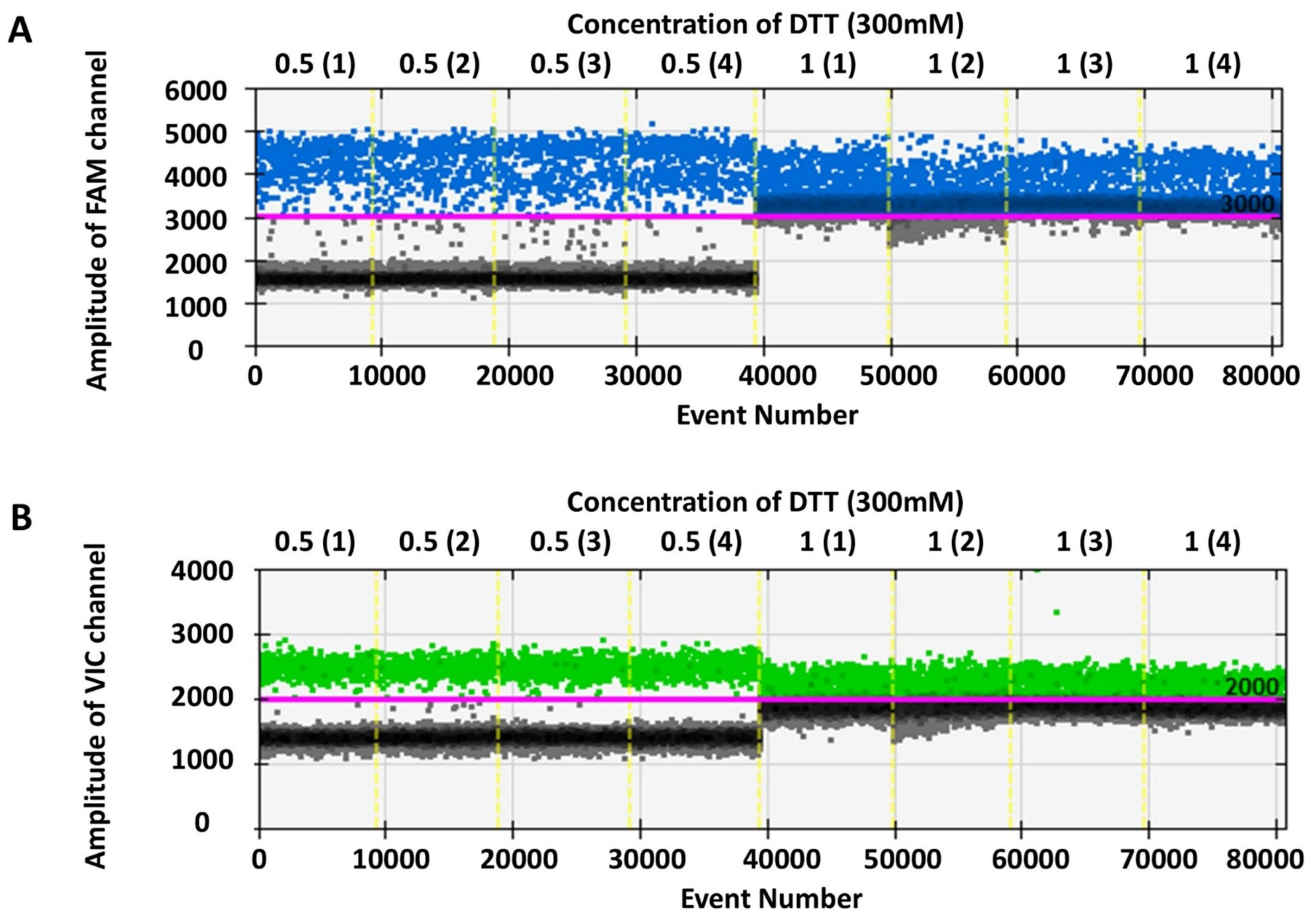
One-dimensional scatter plot comparing 0.5 μL and 1 μL DTT (300 mM) in RT-ddPCR assay. Panels **(A,B)** display the concentration of 300 mM DTT, with 0.5 μL and 1 μL of DTT in the RT-ddPCR system, respectively, 0.5 (1)–(4) and 1 (1)–(4) indicating repeated four times. The y-axis represents the fluorescence amplitude in FAM and VIC channels.

The results comparing the optimal volume of 300 mM DTT in the reaction system, between 0.5 μL and 1 μL, were replicated four times each (see Figure 2). Using of 0.5 μL 300 mM DTT in a 20 μL reaction system enabled clear differentiation between positive and negative droplets. Consequently, 0.5 μL is considered the optimal concentration of 300 mM DTT. Therefore, the final optimized RT-ddPCR reaction system consisted of 20 μL comprising 5 μL SuperMix, 2 μL primer-probe mix, 2 μL reverse transcriptase, 0.5 μL DTT (300 mM), 8.5 μL nuclease-free water, and 2 μL RNA template.

### 3.2 Evaluation of RT-ddPCR sensitivity, specificity, and repeatability

Three strains of SARS-CoV-2, original, Delta, and Omicron, were initially diluted to concentrations of 10^5^, 10^4^, and 10^4^ copies/reaction, respectively, and followed by 2-fold serial dilution for RT-ddPCR analysis. Each dilution underwent 16 repetitions to achieve a 95% positive detection rate. Probit regression analysis was performed on the average copy numbers and positive detection rates of the N and S genes across various dilutions for each strain. The fitted curves demonstrated that the *p* values from Probit analysis for all three strains were <0.05, indicating statistical significance. The LOD for the original strain was 4.26 copies/reaction (95% CI: 3.12–9.89) for the N gene and 3.87 copies/reaction (95% CI: 2.77–7.75) for the S gene. For the Delta strain, the LOD was 4.65 copies/reaction (95% CI: 3.28–9.64) for the N gene and 6.12 copies/reaction (95% CI: 4.33–15.59) for the S gene. For the Omicron strain, the LOD was 4.07 copies/reaction (95% CI: 3.11–6.26) for the N gene and 4.58 copies/reaction (95% CI: 3.43–7.40) for the S gene. Detailed results can be found in Table 3.

**Table 3:** Sensitivity results for three strains of SARS-CoV-2 (original, Delta, Omicron).

Strain	Dilution	Target gene	Average copy number (copies/reaction)	Positive rate (%)	LOD (copies/reaction)	95% CI (copies/reaction)
original	10^–5-16^	N gene	0.58	37.50	4.26	3.12–9.89
	10^–5-8^		2.23	62.50		
	10^–5-4^		2.99	87.50		
	10^–5-2^		8.70	100.00		
	10^−5^		28.75	100.00		
	10^–5-16^	S gene	0.91	50.00	3.87	2.77–7.75
	10^–5-8^		3.79	93.75		
	10^–5-4^		5.79	100.00		
	10^–5-2^		12.26	100.00		
	10^−5^		50.50	100.00		
Delta	10^–4-32^	N gene	0.44	31.25	4.65	3.28–9.64
	10^–4-16^		3.68	87.50		
	10^–4-8^		9.99	100.00		
	10^–4-4^		25.63	100.00		
	10^–4-2^		76.50	100.00		
	10^−4^		189.50	100.00		
	10^–4-32^	S gene	1.61	50.00	6.12	4.33–15.59
	10^–4-16^		5.83	93.75		
	10^–4-8^		12.05	100.00		
	10^–4-4^		26.38	100.00		
	10^–4-2^		88.88	100.00		
	10^−4^		215.88	100.00		
Omicron	10^–4-16^	N gene	0.16	6.25	4.07	3.11–6.26
	10^–4-8^		0.21	12.50		
	10^–4-4^		1.28	62.50		
	10^–4-2^		3.80	87.50		
	10^−4^		11.84	100.00		
	10^–4-16^	S gene	0.15	6.25	4.58	3.43–7.40
	10^–4-8^		0.54	31.25		
	10^–4-4^		1.40	43.75		
	10^–4-2^		4.59	93.75		
	10^−4^		13.83	100.00		

**Figure 3:**
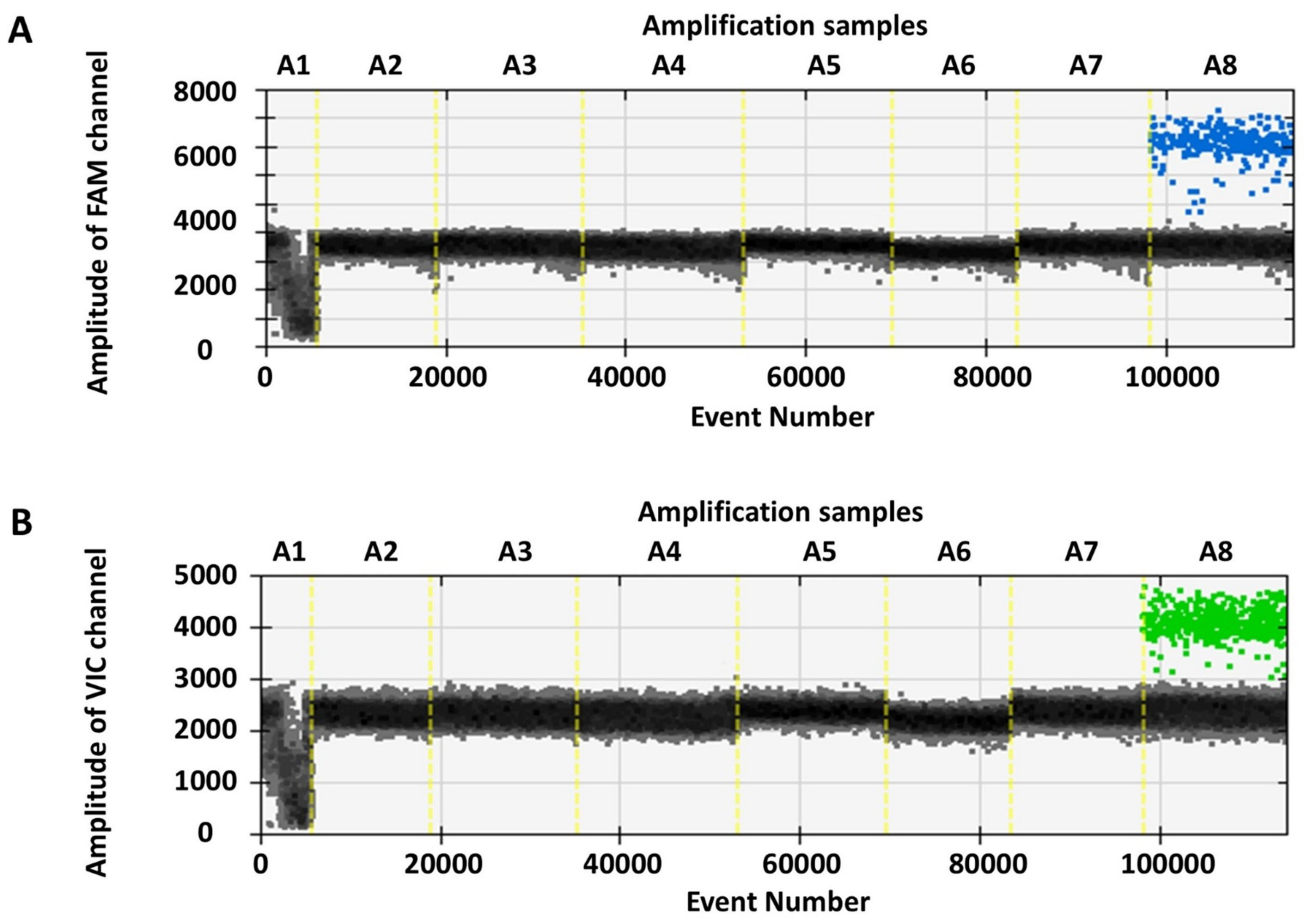
The RT-ddPCR method was used to detect respiratory viruses and SARS-CoV-2 pseudovirus quantitative reference material. In figures **(A,B)**, the x-axis (A1-A8) respectively represents influenza A (H1N1)pdm 09, Victoria lineage of influenza B virus, respiratory syncytial virus subtype A, parainfluenza virus type III, adenovirus type 7, human coronavirus OC43, human coronavirus 229E, and SARS-CoV-2 pseudovirus quantitative reference material, while the y-axis denotes the amplitude of FAM and VIC channels.

The specificity of RT-ddPCR was evaluated with influenza A (H1N1)pdm 09, Victoria lineage of influenza B virus, respiratory syncytial virus subtype A, parainfluenza virus type III, adenovirus type 7, human coronavirus OC43, human coronavirus 229E, and SARS-CoV-2 pseudovirus quantification reference material (see Figure 3). Positive droplets were observed for only the SARS-CoV-2 pseudovirus quantification reference material, whereas no positive detection was observed for the other viruses. This funding indicates that the RT-ddPCR method exhibits specificity for SARS-CoV-2 detection without cross-reacting with other respiratory viruses.

The repeatability evaluation results of SARS-CoV-2 pseudovirus quantitative reference materials using the RT-ddPCR method were presented in Table 4. These results were compared with the target values specified in the instructions: L1: 1 × 10^2^ copies/mL, L2: 1 × 10^3^ copies/mL, L3: 1 × 10^4^ copies/mL, L4: 1 × 10^5^ copies/mL, and L5: 1 × 10^6^ copies/mL. The errors ranged from 1.38 to 11.38%, thus demonstrating consistency between the quantitative results and the target values of the reference materials. For nucleic acid copy numbers >73.50 copies/reaction, the coefficients of variation, calculated from the mean and standard deviation, were all below 10%, thereby indicating that the RT-ddPCR had excellent repeatability within the range of 73.50–7,500 copies/reaction, thus ensuring the reliability experimental outcomes.

**Table 4:** RT-ddPCR detection results of SARS-CoV-2 pseudovirus quantitative reference material.

Number	Target gene	Repetitions	Average copy number (copies/reaction)	Standard deviation	Coefficient of variation (%)
L1	N gene	16	1.76	0.75	42.69
	S gene		1.26	0.76	60.51
L2	N gene	16	8.21	2.84	34.61
	S gene		8.00	2.35	29.39
L3	N gene	16	73.50	7.47	10.16
	S gene		76.88	7.55	9.82
L4	N gene	16	769.25	44.52	5.79
	S gene		789	44.77	5.67
L5	N gene	16	7,535	461.87	6.13
	S gene		7,090	434.57	6.13

### 3.3 Comparison of RT-ddPCR and RT-qPCR detection among SARS-CoV-2 variants

**Figure 4:**
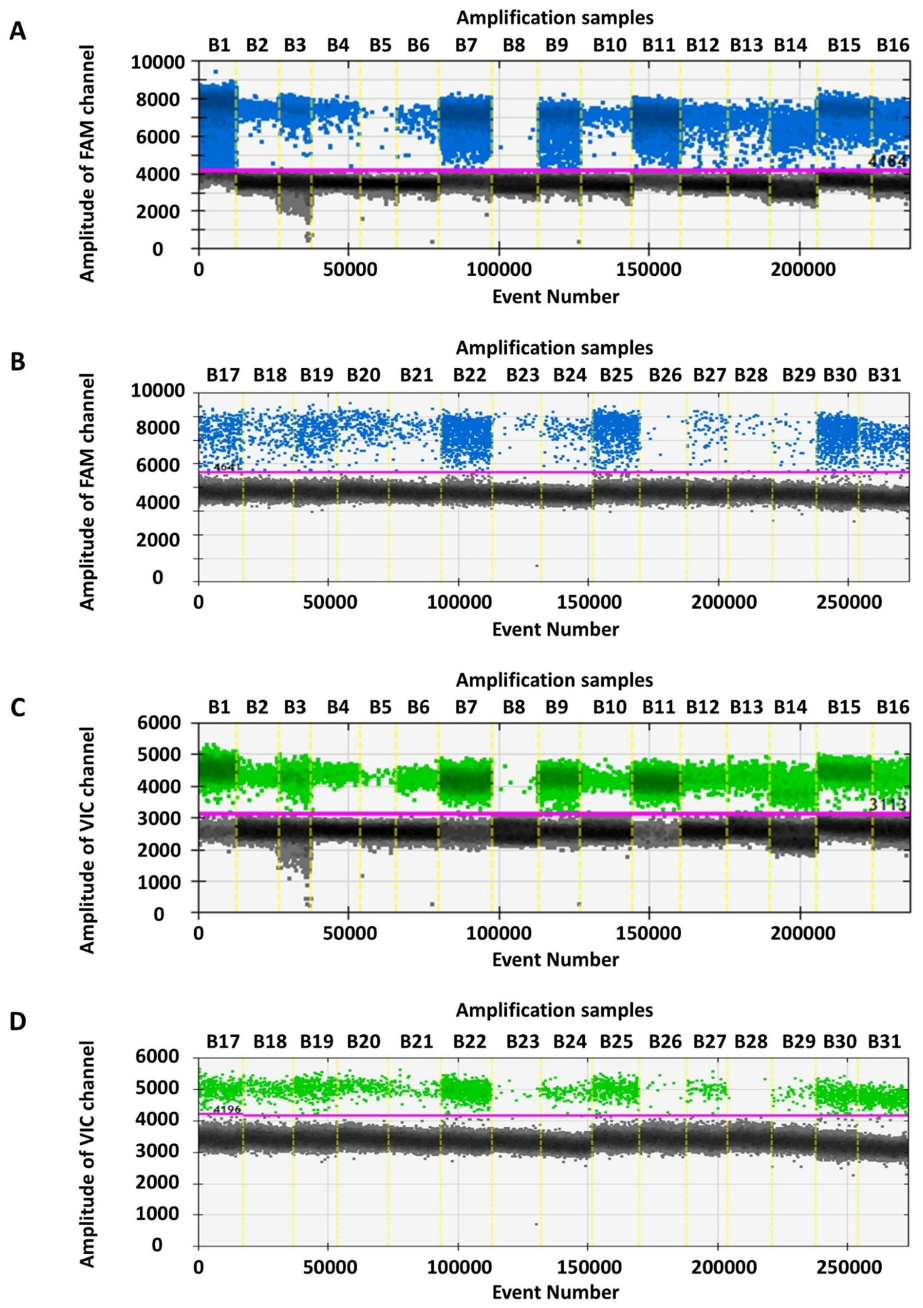
Detection results of the original strain and thirty SARS-CoV-2 variants by RT-ddPCR method. In figures **(A,B)**, the x-axis (B1-B31) respectively represent original, Alpha (B.1.1.7), Delta (B.1.617.2), and Omicron (BA.1.1, BA.2.12.1, BA.2.3, XBB.1.5.4, XBB.1.9.2, EG.5.1, EG.5.1.1, HK.3, XBB.1.16, FU.1, XBB.1.22, BA.4.1, BA.5.2, BF.7, BA.5.2.48, DY.2, BQ.1.1, BF.7.14, JN.1, JN.1.4.5, LB.1.2, JN.1.16, KP.2, KP.3.1.1, JN.1.67.1, XDV.1, XDV.1.5, and NB.1), and the y-axis represent the amplitude of FAM channel. In figures **(C,D)**, the x-axis (B1-B31) respectively represent the original strain and thirty variants, and the y-axis represent the amplitude of VIC channel.

The RT-ddPCR results for the original strain and 30 variants of SARS-CoV-2 revealed copy numbers ranging from 3 to 46,010 copies/reaction for the N gene, and from 1.4 to 54,300 copies/reaction for the S gene. Correspondingly, the N gene CT values in RT-qPCR ranged from 37.69 to 23.55, whereas the S gene CT values ranged from 37.47 to 22.51. These findings are detailed in Table 5 and illustrated in Figure 4. The consistency between the results of the two methods across the SARS-CoV-2 variants suggested that RT-ddPCR was well-suited to detection of SARS-CoV-2 variants.

**Table 5:** Detection results of SARS-CoV-2 variants by RT-ddPCR and RT-qPCR.

Lineage	Sublineage		N gene		S gene	
			CT value	Copy number (copies/reaction)	CT value	Copy number (copies/reaction)
Original			24.27	46,010	23.47	52,700
Alpha	B.1.1.7		30.94	232	30.11	322
Delta	B.1.617.2		28.71	2,335	27.90	2,870
Omicron	BA.1	BA.1.1	31.28	231	29.91	448
	BA.2	BA.2.12.1	35.63	19.9	34.74	50
		BA.2.3	34.93	188	33.18	403
		JN.1	28.68	555	27.81	600
		JN.1.4.5	34.19	17	34.35	6.8
		LB.1.2	31.19	68	30.55	70
		KP.2	35.30	4.6	33.76	7.2
		KP.3.1.1	31.84	55	30.96	61
		JN.1.16	29.22	518	29.61	210
		JN.1.67.1	32.84	13	34.70	1.4
	BA.4	BA.4.1	26.05	6,560	25.71	6,830
	BA.5	BA.5.2	28.16	1,456	28.26	1,102
		BA.5.2.48	31.32	96	30.65	96
		BF.7	29.95	223	29.43	234
		BF.7.14	32.54	57	31.87	44
		DY.2	29.99	233	29.50	234
		BQ.1.1	31.05	126	30.64	101
	XBB	XBB.1.5.4	24.18	23,090	23.49	34,620
		XBB.1.9.2	37.69	3	37.47	6.8
		EG.5.1	28.24	4,770	26.64	10,110
		EG.5.1.1	33.02	222	31.98	330
		HK.3	23.55	39,520	22.51	54,300
		XBB.1.16	28.96	713	27.73	1,165
		FU.1	29.52	494	28.23	1,130
		XBB.1.22	31.03	1,054	29.87	1780
	XDV	XDV.1	32.64	23	31.93	30
		XDV.1.5	28.62	603	29.00	281
		NB.1	29.62	186	28.76	280

### 3.4 Quantitative detection results of clinical and wastewater samples by RT-ddPCR

We compared the established RT-ddPCR method with RT-qPCR in the detection of 148 clinical specimens and 50 wastewater samples. The RT-ddPCR analysis consistently detected more than 10,000 effective droplets per sample, thus ensuring data reliability. Among the 148 clinical specimens, both RT-ddPCR and RT-qPCR identified 128 positives for dual gene targets and 20 negatives, and the positive rate were 86.49% between two methods. There were 146 samples with consistent results, with a concordance rate of 98.65% and a Kappa value of 0.94. At 95% confidence interval, the sensitivity was 99.22% (95.02–99.96%) and the specificity was 95.00% (73.06–99.74%). RT-ddPCR quantification results ranged from 1.8 to 61,600 copies/reaction for the N gene and from 1.4 to 60,600 copies/reaction for the S gene. Corresponding RT-qPCR CT values ranged from 18.90 to 38.72 for the N gene and 18.51 to 38.86 for the S gene (see Supplementary Table 1).

In the analysis of low-concentration wastewater samples, RT-ddPCR detected 50 positives, whereas RT-qPCR identified 21 were positive for both the N and S genes, 25 were positive for the S gene, and 4 were negative. RT-ddPCR quantification results ranged from 0.6 to 163 copies/reaction for the N gene, and from 0.6 to 136 copies/reaction for the S gene. In contrast, in RT-qPCR, the CT values ranged from 37.92 to 32.01 for the N gene, and from 38.16 to 30.33 for the S gene. Overall, RT-ddPCR had slightly superior quantification performance to that of RT-qPCR in detecting low viral loads (see Table 6).

**Table 6:** Detection of SARS-CoV-2 in wastewater samples by RT-ddPCR and RT-qPCR.

Sample	Target gene	CT value	Copy number (copies/reaction)	Sample	Target gene	CT value	Copy number (copies/reaction)
1	N gene	35.36	8.3	26	N gene	-	1.2
	S gene	34.47	11.3		S gene	38.01	0.6
2	N gene	36.32	5.3	27	N gene	-	3.6
	S gene	35.56	0.6		S gene	34.04	4.7
3	N gene	37.92	1.9	28	N gene	-	3.7
	S gene	37.86	1.2		S gene	35.02	6.2
4	N gene	36.67	4.8	29	N gene	-	5.9
	S gene	35.56	1.8		S gene	34.60	6.3
5	N gene	33.34	57	30	N gene	-	2.3
	S gene	32.61	53		S gene	36.55	1.7
6	N gene	36.54	2.4	31	N gene	-	3.5
	S gene	35.50	3.6		S gene	34.10	4.6
7	N gene	36.44	3.5	32	N gene	-	4.6
	S gene	35.84	7.5		S gene	34.28	4.6
8	N gene	36.85	22	33	N gene	-	5.8
	S gene	34.17	12.8		S gene	33.96	5.8
9	N gene	35.72	7.4	34	N gene	-	3.6
	S gene	35.22	4.3		S gene	33.13	6
10	N gene	34.29	42	35	N gene	-	4.3
	S gene	32.43	42		S gene	34.81	8.7
11	N gene	34.04	70	36	N gene	-	0.6
	S gene	32.27	54		S gene	35.97	0.6
12	N gene	32.08	163	37	N gene	-	5
	S gene	30.50	136		S gene	34.25	8.2
13	N gene	35.31	122	38	N gene	-	3.2
	S gene	32.63	105		S gene	34.76	1.3
14	N gene	34.47	104	39	N gene	-	1.9
	S gene	32.38	97		S gene	35.46	1.9
15	N gene	33.68	99	40	N gene	-	3.4
	S gene	31.97	76		S gene	35.82	4
16	N gene	37.11	5.8	41	N gene	-	8.1
	S gene	36.01	2.2		S gene	34.00	2.9
17	N gene	34.46	18.2	42	N gene	-	8
	S gene	33.21	32		S gene	34.47	8
18	N gene	37.14	6.2	43	N gene	-	3.2
	S gene	34.76	3.6		S gene	34.53	3.2
19	N gene	36.42	1.6	44	N gene	-	1.3
	S gene	37.13	1.2		S gene	36.24	0.6
20	N gene	32.82	26	45	N gene	-	1.2
	S gene	31.89	54		S gene	34.09	1.2
21	N gene	32.01	90	46	N gene	-	4.3
	S gene	30.33	126		S gene	34.37	3.7
22	N gene	-	1.2	47	N gene	-	0.7
	S gene	38.16	2.6		S gene	-	2
23	N gene	-	4.1	48	N gene	-	0.6
	S gene	32.58	7.1		S gene	-	3.9
24	N gene	-	1.3	49	N gene	-	0.6
	S gene	35.72	2.6		S gene	-	1.2
25	N gene	-	3	50	N gene	-	3.9
	S gene	36.02	3		S gene	-	2.8

## 4 Discussion

Since the onset of the COVID-19 pandemic, SARS-CoV-2 has been mutating and spreading globally, thus posing a considerable public health threat. Traditional RT-qPCR methods often fail to accurately quantify viral copies, and may potentially fail to detect low concentrations of nucleic acids. Developing a more precise, efficient, and quantifiable detection method is imperative for effective epidemic prevention and control.

We developed an RT-ddPCR method that enhances accuracy by quantifying SARS-CoV-2 variants with dual primers and probes targeting the N and S genes. SARS-CoV-2 N protein has evolutionary conservation, which was used for great diagnostic marker (Eltayeb et al., 2024). Notably, the SARS-CoV-2 S glycoprotein is composed of two subunits, S1 and S2. In the prefusion state, the S1 subunit mediates binding to the host cell receptor angiotensin converting enzyme 2 (ACE2), while the S2 subunit drives viral envelope fusion with the host membrane (Wrapp et al., 2020). Mutations in SARS-CoV-2 variants predominantly localize to the receptor-binding domain (RBD) of the S1 subunit (Walls et al., 2020). As the primary target for neutralizing antibodies, the S protein elicits potent humoral immunity, with the RBD harboring the dominant neutralizing epitopes responsible for over 90% of neutralizing activity (Jackson et al., 2022). In this study, our forward primer (5′-3′, 2986–3009), reverse primer (5′-3′, 3036–3060), and probe (5′-3′, 3011–3029) of S gene were targeting the central helices (CH) in relatively high conservation region, demonstrating the universal detection efficacy across SARS-CoV-2 variants. Highly conserved primer-probe sets are essential for the detection of SARS-CoV-2 variants.

Unlike traditional methods, RT-ddPCR does not rely on standard curves, thereby enabling direct quantification of nucleic acid concentrations and demonstrating superior sensitivity (Huggett et al., 2015; Park et al., 2021). Tao has highlighted that ddPCR has greater sensitivity than RT-qPCR in detecting low viral loads, and has benefits of requiring minimal nucleic acid amounts, without a need for repeated sampling or extensive reagents (Suo et al., 2020). Herein, we conducted sensitivity experiments on three SARS-CoV-2 strains and achieved a lowest detection limit of <6.12 copies/reaction with a 95% positive detection rate, thus validating our method’s high sensitivity in the detection of samples with low viral load. In terms of specificity, our RT-ddPCR method showed no cross-reactivity with common respiratory viruses such as influenza A and B viruses, respiratory syncytial virus, parainfluenza virus, and other coronaviruses. Additionally, repeatability studies using SARS-CoV-2 pseudovirus RNA revealed a coefficient of variation <10% at nucleic acid concentrations ranging from 73.50 to 7,500 copies/reaction, in agreement with the highly reproducible results observed in various laboratory tests by Whale et al. (2017). Furthermore, RT-ddPCR accurately quantified SARS-CoV-2 variants, thereby underscoring its excellent specificity. Compared with RT-qPCR, RT-ddPCR provided advantages in precise quantification and accuracy in detecting clinical and environmental samples.

Despite its advantages, RT-ddPCR had several limitations. Quantification accuracy may be compromised at high target concentrations (≥10^5^ copies/reaction) due to saturation effects, reducing the confidence in detection (Quan et al., 2018). In such cases, pre-dilution experiments should be conducted before detection. Additionally, RT-ddPCR necessitated higher standards for instruments, equipment, and experimental personnel, thus contributing to its lower adoption than other methods (Kojabad et al., 2021).

In conclusion, this study established an RT-ddPCR detection method that accurately quantified low concentrations of SARS-CoV-2 variants, exhibiting robust specificity, high sensitivity, and excellent repeatability. This method was well-suited to early clinical detection of SARS-CoV-2 infections and tracing viral presence in the environment. We believe that this method may be applied to provide valuable insights in clinical diagnosis and treatment, thus ultimately mitigating the risk and effects of viral transmission.

## Data Availability

The original contributions presented in the study are included in the article/Supplementary material, further inquiries can be directed to the corresponding authors.
