# Supplementary material for: Droplet digital RT-PCR method for SARS-CoV-2 variants detection in clinical and wastewater samples
Source: Front Microbiol. 2025 Jul 3;16:1635733. doi: 10.3389/fmicb.2025.1635733 (PMC12267190; doi:10.3389/fmicb.2025.1635733)
Supplement: Supplementary file 1 [file Table_1.DOCX]

Supplementary Material

**Supplementary Table 1.** Detection of SARS-CoV-2 in 148 clinical specimens by RT-ddPCR and RT-qPCR

| Sample | Target gene | CT value | Copy number (copies/  reaction) | Sample | Target gene | CT value | Copy number (copies/  reaction) |
| --- | --- | --- | --- | --- | --- | --- | --- |
| 1 | N gene | 22.23 | 50460 | 75 | N gene | 24.47 | 43880 |
|  | S gene | 21.91 | 52640 |  | S gene | 23.69 | 44700 |
| 2 | N gene | 27.87 | 3882 | 76 | N gene | 31.99 | 574 |
|  | S gene | 27.20 | 4380 |  | S gene | 31.12 | 598 |
| 3 | N gene | 27.49 | 6860 | 77 | N gene | 34.01 | 148 |
|  | S gene | 27.08 | 6900 |  | S gene | 32.99 | 164 |
| 4 | N gene | 28.96 | 2840 | 78 | N gene | 32.76 | 398 |
|  | S gene | 28.40 | 3180 |  | S gene | 31.99 | 430 |
| 5 | N gene | 27.63 | 8740 | 79 | N gene | 24.19 | 42060 |
|  | S gene | 27.53 | 9080 |  | S gene | 24.75 | 42400 |
| 6 | N gene | 27.78 | 5800 | 80 | N gene | 25.52 | 33420 |
|  | S gene | 27.61 | 6500 |  | S gene | 25.35 | 35400 |
| 7 | N gene | 30.27 | 990 | 81 | N gene | 22.57 | 35220 |
|  | S gene | 29.93 | 1182 |  | S gene | 21.50 | 36520 |
| 8 | N gene | 27.00 | 11420 | 82 | N gene | 22.67 | 33780 |
|  | S gene | 26.58 | 13520 |  | S gene | 21.67 | 34960 |
| 9 | N gene | 25.85 | 36240 | 83 | N gene | 21.16 | 51940 |
|  | S gene | 25.96 | 38640 |  | S gene | 20.29 | 54000 |
| 10 | N gene | 31.64 | 366 | 84 | N gene | 26.36 | 20800 |
|  | S gene | 31.07 | 488 |  | S gene | 25.93 | 22960 |
| 11 | N gene | 25.09 | 27880 | 85 | N gene | 22.65 | 44680 |
|  | S gene | 24.23 | 30900 |  | S gene | 21.74 | 45420 |
| 12 | N gene | 22.72 | 43520 | 86 | N gene | 20.62 | 59400 |
|  | S gene | 22.65 | 46200 |  | S gene | 20.07 | 60200 |
| 13 | N gene | 20.43 | 53060 | 87 | N gene | 21.96 | 51300 |
|  | S gene | 19.94 | 59400 |  | S gene | 21.49 | 59200 |
| 14 | N gene | 24.15 | 31740 | 88 | N gene | 19.65 | 56200 |
|  | S gene | 24.37 | 33460 |  | S gene | 18.97 | 59260 |
| 15 | N gene | 20.75 | 52700 | 89 | N gene | 19.89 | 57600 |
|  | S gene | 20.30 | 58420 |  | S gene | 19.81 | 58800 |
| 16 | N gene | 18.90 | 61600 | 90 | N gene | 33.36 | 376 |
|  | S gene | 19.28 | 57800 |  | S gene | 31.98 | 360 |
| 17 | N gene | 25.98 | 20120 | 91 | N gene | 34.00 | 378 |
|  | S gene | 25.92 | 21780 |  | S gene | 31.91 | 398 |
| 18 | N gene | 24.55 | 34280 | 92 | N gene | 24.20 | 47260 |
|  | S gene | 24.39 | 34580 |  | S gene | 23.23 | 47680 |
| 19 | N gene | 20.71 | 42480 | 93 | N gene | 27.45 | 7260 |
|  | S gene | 20.75 | 42920 |  | S gene | 27.24 | 7900 |
| 20 | N gene | 23.41 | 29040 | 94 | N gene | 29.05 | 3780 |
|  | S gene | 22.93 | 30140 |  | S gene | 28.65 | 3520 |
| 21 | N gene | 33.80 | 248 | 95 | N gene | 19.40 | 58200 |
|  | S gene | 32.81 | 324 |  | S gene | 18.51 | 60600 |
| 22 | N gene | 25.32 | 36540 | 96 | N gene | 30.90 | 164 |
|  | S gene | 25.17 | 37440 |  | S gene | 31.25 | 78 |
| 23 | N gene | 28.18 | 7020 | 97 | N gene | 28.72 | 108 |
|  | S gene | 27.95 | 8180 |  | S gene | 30.20 | 84 |
| 24 | N gene | 25.58 | 35280 | 98 | N gene | 34.30 | 4.6 |
|  | S gene | 24.53 | 35420 |  | S gene | 35.56 | 1.6 |
| 25 | N gene | 24.30 | 30160 | 99 | N gene | 32.75 | 9.8 |
|  | S gene | 24.17 | 33540 |  | S gene | 34.37 | 2.8 |
| 26 | N gene | 28.63 | 4580 | 100 | N gene | 29.05 | 70 |
|  | S gene | 27.89 | 4800 |  | S gene | 29.57 | 68 |
| 27 | N gene | 32.40 | 386 | 101 | N gene | 30.36 | 34 |
|  | S gene | 31.53 | 468 |  | S gene | 31.90 | 24.4 |
| 28 | N gene | 29.74 | 2360 | 102 | N gene | 36.63 | 13.6 |
|  | S gene | 28.96 | 2500 |  | S gene | 38.32 | 8.6 |
| 29 | N gene | 27.23 | 10500 | 103 | N gene | 35.07 | 6.8 |
|  | S gene | 27.08 | 12900 |  | S gene | 38.09 | 5 |
| 30 | N gene | 27.54 | 11340 | 104 | N gene | 24.45 | 2480 |
|  | S gene | 26.63 | 12360 |  | S gene | 24.74 | 2340 |
| 31 | N gene | 26.60 | 22300 | 105 | N gene | 36.14 | 5.6 |
|  | S gene | 25.97 | 23720 |  | S gene | 37.32 | 9.2 |
| 32 | N gene | 33.22 | 474 | 106 | N gene | 35.69 | 28 |
|  | S gene | 32.15 | 430 |  | S gene | 37.12 | 6.2 |
| 33 | N gene | 34.40 | 402 | 107 | N gene | 33.22 | 24 |
|  | S gene | 34.03 | 400 |  | S gene | 33.73 | 46 |
| 34 | N gene | 29.76 | 2680 | 108 | N gene | 29.08 | 760 |
|  | S gene | 28.69 | 2740 |  | S gene | 29.98 | 722 |
| 35 | N gene | 29.38 | 2680 | 109 | N gene | 30.87 | 124 |
|  | S gene | 28.36 | 2740 |  | S gene | 31.36 | 118 |
| 36 | N gene | 25.72 | 29040 | 110 | N gene | 36.57 | 30 |
|  | S gene | 25.26 | 30220 |  | S gene | 36.29 | 12 |
| 37 | N gene | 29.72 | 1650 | 111 | N gene | 31.78 | 5.6 |
|  | S gene | 28.77 | 2080 |  | S gene | 33.10 | 2.4 |
| 38 | N gene | 24.74 | 31560 | 112 | N gene | 33.36 | 12 |
|  | S gene | 24.29 | 35020 |  | S gene | 33.99 | 14 |
| 39 | N gene | 27.98 | 10520 | 113 | N gene | 38.68 | 5.4 |
|  | S gene | 26.96 | 11740 |  | S gene | 38.14 | 9.8 |
| 40 | N gene | 26.08 | 30640 | 114 | N gene | 36.63 | 8.6 |
|  | S gene | 25.58 | 31780 |  | S gene | 37.16 | 6.8 |
| 41 | N gene | 24.24 | 43480 | 115 | N gene | 37.29 | 1.8 |
|  | S gene | 23.75 | 45240 |  | S gene | 37.96 | 3.4 |
| 42 | N gene | 22.96 | 50560 | 116 | N gene | 31.61 | 78 |
|  | S gene | 22.51 | 51040 |  | S gene | 31.81 | 64 |
| 43 | N gene | 33.30 | 204 | 117 | N gene | 34.93 | 12.5 |
|  | S gene | 32.03 | 228 |  | S gene | 37.18 | 5.0 |
| 44 | N gene | 23.81 | 48800 | 118 | N gene | 36.42 | 8.4 |
|  | S gene | 23.37 | 50580 |  | S gene | 36.81 | 5.8 |
| 45 | N gene | 36.37 | 18.2 | 119 | N gene | 27.23 | 274 |
|  | S gene | 35.73 | 26 |  | S gene | 30.12 | 148 |
| 46 | N gene | 23.31 | 45820 | 120 | N gene | 35.84 | 14 |
|  | S gene | 22.95 | 48780 |  | S gene | 36.52 | 2.4 |
| 47 | N gene | 28.58 | 3380 | 121 | N gene | 32.20 | 9.6 |
|  | S gene | 28.44 | 3540 |  | S gene | 32.61 | 11 |
| 48 | N gene | 31.62 | 580 | 122 | N gene | 31.39 | 11.6 |
|  | S gene | 31.43 | 592 |  | S gene | 32.62 | 20.2 |
| 49 | N gene | 29.12 | 12140 | 123 | N gene | 36.90 | 8.2 |
|  | S gene | 28.43 | 12820 |  | S gene | 38.23 | 1.6 |
| 50 | N gene | 23.13 | 52060 | 124 | N gene | 30.96 | 38 |
|  | S gene | 22.92 | 54600 |  | S gene | 32.73 | 23.2 |
| 51 | N gene | 28.03 | 8280 | 125 | N gene | 35.87 | 11.6 |
|  | S gene | 27.10 | 8440 |  | S gene | 36.42 | 8.4 |
| 52 | N gene | 21.88 | 52620 | 126 | N gene | 33.76 | 19.6 |
|  | S gene | 20.98 | 55600 |  | S gene | 34.89 | 12.8 |
| 53 | N gene | 20.53 | 50020 | 127 | N gene | 36.31 | 4.8 |
|  | S gene | 19.94 | 50160 |  | S gene | 37.34 | 3.2 |
| 54 | N gene | 27.75 | 7140 | 128 | N gene | 38.72 | 0 |
|  | S gene | 27.06 | 7270 |  | S gene | 38.86 | 0 |
| 55 | N gene | 27.56 | 7960 | 129 | N gene | - | 2.8 |
|  | S gene | 26.96 | 8300 |  | S gene | - | 1.4 |
| 56 | N gene | 23.67 | 45260 | 130 | N gene | - | 0 |
|  | S gene | 23.40 | 46840 |  | S gene | - | 0 |
| 57 | N gene | 21.94 | 42900 | 131 | N gene | - | 0 |
|  | S gene | 21.49 | 43920 |  | S gene | - | 0 |
| 58 | N gene | 23.58 | 34020 | 132 | N gene | - | 0 |
|  | S gene | 22.96 | 36720 |  | S gene | - | 0 |
| 59 | N gene | 24.97 | 24660 | 133 | N gene | - | 0 |
|  | S gene | 24.62 | 25280 |  | S gene | - | 0 |
| 60 | N gene | 19.13 | 46920 | 134 | N gene | - | 0 |
|  | S gene | 18.54 | 48400 |  | S gene | - | 0 |
| 61 | N gene | 24.33 | 20900 | 135 | N gene | - | 0 |
|  | S gene | 24.97 | 21180 |  | S gene | - | 0 |
| 62 | N gene | 32.54 | 278 | 136 | N gene | - | 0 |
|  | S gene | 31.86 | 308 |  | S gene | - | 0 |
| 63 | N gene | 28.77 | 6980 | 137 | N gene | - | 0 |
|  | S gene | 27.59 | 7120 |  | S gene | - | 0 |
| 64 | N gene | 26.88 | 12460 | 138 | N gene | - | 0 |
|  | S gene | 26.21 | 13940 |  | S gene | - | 0 |
| 65 | N gene | 22.76 | 41600 | 139 | N gene | - | 0 |
|  | S gene | 22.60 | 44400 |  | S gene | - | 0 |
| 66 | N gene | 22.43 | 44580 | 140 | N gene | - | 0 |
|  | S gene | 21.20 | 45940 |  | S gene | - | 0 |
| 67 | N gene | 34.00 | 236 | 141 | N gene | - | 0 |
|  | S gene | 32.79 | 250 |  | S gene | - | 0 |
| 68 | N gene | 29.72 | 2840 | 142 | N gene | - | 0 |
|  | S gene | 29.03 | 2860 |  | S gene | - | 0 |
| 69 | N gene | 33.93 | 344 | 143 | N gene | - | 0 |
|  | S gene | 31.97 | 398 |  | S gene | - | 0 |
| 70 | N gene | 30.37 | 2480 | 144 | N gene | - | 0 |
|  | S gene | 29.07 | 2640 |  | S gene | - | 0 |
| 71 | N gene | 28.35 | 8820 | 145 | N gene | - | 0 |
|  | S gene | 27.26 | 8900 |  | S gene | - | 0 |
| 72 | N gene | 34.23 | 188 | 146 | N gene | - | 0 |
|  | S gene | 33.09 | 234 |  | S gene | - | 0 |
| 73 | N gene | 31.39 | 1092 | 147 | N gene | - | 0 |
|  | S gene | 30.15 | 1008 |  | S gene | - | 0 |
| 74 | N gene | 28.44 | 8080 | 148 | N gene | - | 0 |
|  | S gene | 27.75 | 8300 |  | S gene | - | 0 |
